# Excitation-Contraction Coupling between Human Atrial Myocytes with Fibroblasts and Stretch Activated Channel Current: A Simulation Study

**DOI:** 10.1155/2013/238676

**Published:** 2013-08-13

**Authors:** Heqing Zhan, Ling Xia

**Affiliations:** Department of Biomedical Engineering, Zhejiang University, Hangzhou 310027, China

## Abstract

Myocytes have been regarded as the main objectives in most cardiac modeling studies and attracted a lot of attention. Connective tissue cells, such as fibroblasts (Fbs), also play crucial role in cardiac function. This study proposed an integrated myocyte-*I*_sac_-Fb electromechanical model to investigate the effect of Fbs and stretch activated ion channel current (*I*_sac_) on cardiac electrical excitation conduction and mechanical contraction. At the cellular level, an active Fb model was coupled with a human atrial myocyte electrophysiological model (including *I*_sac_) and a mechanical model. At the tissue level, electrical excitation conduction was coupled with an elastic mechanical model, in which finite difference method (FDM) was used to solve the electrical excitation equations, while finite element method (FEM) was used for the mechanics equations. The simulation results showed that Fbs and *I*_sac_ coupling caused diverse effects on action potential morphology during repolarization, depolarized the resting membrane potential of the human atrial myocyte, slowed down wave propagation, and decreased strains in fibrotic tissue. This preliminary simulation study indicates that Fbs and *I*_sac_ have important implications for modulating cardiac electromechanical behavior and should be considered in future cardiac modeling studies.

## 1. Introduction

Heart is considered as a composite material consisting of myocytes, Fbs, endothelial, vascular smooth muscle, neuronal cells, and fluids [1, 2]. The division and interaction of these cells and fluids keep the heart working efficiently, which is performed by a well-ordered interplay between cardiac electrophysiology, excitation propagation, and force development. The process from the electrical excitation of the myocyte to mechanical contraction is referred to as cardiac excitation-contraction coupling (ECC). Conversely, changing the cardiac mechanical environment to alter electrical activity is referred to as mechanoelectric feedback (MEF) [3]. Investigating the multiphysics and multiscale heart system involves extremely difficult experiments in order to directly observe and manipulate the process that underlies cardiac electrical and mechanical activity. To overcome these experimental challenges, some sophisticated mathematical models have been developed to gain a better insight [4–9].

 To study cardiac ECC, many electromechanical (EM) models, from the molecular level of myofilaments (MFs) to the anatomy of the organs, have been developed under both normal and pathological situations [6, 10–14]. At the subcellular level, actin-myosin interaction and Ca-based activation are represented by MF models. Cellular reconstruction of electrophysiology and Ca handling are then coupled with MF models to produce EM cell models. At the organ level, electrical component is solved as a reaction-diffusion system and mechanical component is described by equations of continuum mechanics [4].

 For cardiac MEF, the main focus of published studies has been placed on the function of stretch activated channels (SACs) in various noncardiac tissues [15, 16] as well as in the heart [17, 18]. The electrophysiologic effects of pulsatile stretch, stretch generated by increased preload and afterload, and acute static mechanical stretch can be explained by SAC current (*I*_sac_).

 The published models of ECC and MEF mainly described the properties of cardiac myocytes. However, the vasculature and connective tissue cells [19] have not been considered. Recently, it has been revealed that Fbs are numerously present in cardiac tissues with much smaller size than that of myocytes [20], and they affect the restitution properties of cardiac tissues, especially during the process of ageing and in various cardiac diseases [21–23]. Once the percentage of Fbs in the heart increases up to 10–35%, a remodeling of cardiac structure occurs, which increases muscle stiffness and reduces the coupling between adjacent muscle fiber bundles [24]. The functional roles of Fbs on cardiac electrical and mechanical activities have attracted more and more interests. Both experimental and computational studies [25–27] have confirmed that increased Fb population could lead to nonmonotonic changes in the conduction velocity and implicated that the effects of Fbs on cardiac electrophysiology and mechanics are worthy of further investigation.

 Although the myocytes have been regarded as the main objectives for the cardiac electrophysiological, mechanical, and EM models [5–8], the role of Fbs has also been investigated recently using the models. By including the Fbs in the cardiac electrophysiological simulations [1, 27–29], it has been reported that myocyte-Fb coupling modulated action potential (AP) morphology and action potential duration (APD). However, to our best knowledge, due to the lack of experimental data and accurate models of Fb mechanics, the myocytes and Fbs have not yet been integrated to investigate their electromechanics interaction. The aim of this study was to propose an integrated myocyte-*I*_sac_-Fb electromechanical model to investigate the effect of Fbs and stretch activated ion channel current (*I*_sac_) on cardiac electrical excitation conduction and mechanical contraction. The proposed model integrated the coupling between the Fb model [30] and the myocardial electrophysiological model [31] (including *I*_sac_ [32]) and mechanical model [33] at the cellular level, with excitation conduction and elastic mechanics [34] at the tissue level. The developed model was validated by comparing the simulated results of excitation conduction and mechanical properties to those from previous electromechanical coupling models. Electromechanical models of central point stimulus and strain maps were illustrated to investigate the effects of Fbs and *I*_sac_ on cardiac excitation conduction and mechanical contraction.

## 2. Materials and Methods

The framework of the coupled myocyte-*I*_sac_-Fb model includes two parts. The first part includes the electrophysiological and mechanical models of myocytes and Fbs at the cellular level. The cell model stems from the Courtemanche-Ramirez-Nattel (CRN) model of the human atrial AP [30] (including *I*_sac_ equations described by Kuijpers et al. [31]), the Rice mechanical model [32], and the Maleckar model of atrial Fbs [33]. The second part includes the models of excitation conduction and contraction of the Fb-myocyte coupling (FMC) at the tissue level. The tissue model includes cardiac excitation conduction and finite deformation, described by the Nash model [34]. Details of the individual components and their modification from published models are described as follows.

### 2.1. Myocyte-*I*_sac_-Fb Electrophysiological Modeling at the Cellular Level

#### 2.1.1. Electrophysiological Model of the Human Atrial Myocyte and *I*_sac_

The CRN model was employed in this study, which can accurately represent atrial AP dynamics [30]. This computationally efficient model was based on the experimental data and reproduced various AP behaviors. Equations of *I*_sac_ were described by Kuijpers et al. in [31]. In their model, the amount of *I*_sac_ was related to local stretch and can be changed during contraction.

#### 2.1.2. Electrophysiological Model of Atrial Fbs

For the electrophysiological model of atrial Fbs, the active 1 model described by Maleckar et al. was applied in our study [33]. 

#### 2.1.3. Cell-Cell Electrophysiological Coupling

The electrophysiological FMC is expressed as [33]

(1) dVmyodt =−(1/Cm,myo)[Imyo(Vmyo,t)+Istim+∑i=1:nGgap⁡(Vmyo−VFb)]dVFbdt=−(1/Cm,Fb)[IFb(VFb,t)−Igap⁡],

where *V*_myo_ and *V*_Fb_ are the transmembrane potential of the human atrial myocyte and the Fb, respectively, *C*_*m*,myo_ and *C*_*m*,Fb_ are the membrane capacitance of the myocyte and Fb, respectively, and *I*_myo_ and *I*_Fb_ are the net membrane current of the myocyte and Fb, respectively. *I*_stim_ is the stimulus current applied to the myocyte membrane, and *I*_gap⁡_ is the current that flows through the gap junction between the myocyte and each Fb. *G*_gap⁡_ represents the gap-junctional conductance.

### 2.2. Myocyte-*I*_sac_-Fb Mechanical Modeling at the Cellular Level: The Ca^2+^-Force Relation

To simulate the mechanics of myocyte-*I*_sac_-Fb model, the Ca^2+^-force relation in myocyte and Fb was considered. In the present study, the computationally efficient Rice mechanical model was applied for the myocyte [32], which included phenomenological representations of both regulatory unit (RU)-RU cooperative interactions and a three-state model of crossbridge attachment and distortion [4]. In our study, this MF model was coupled to the CRN model by using the Ca concentration from the CRN model as the input to the MF model. 

To the best of our knowledge, there has been no well-defined mathematical model to describe the tension in Fb. The main reasons are as follows. Firstly, there is lack of experimental data due to the small size of individual Fbs [35]. Secondly, Ca^2+^ current, as a control factor of active tension, has not been identified for membrane ionic currents in Fb [1, 33, 36, 37]. The active force of Fb was therefore ignored at the cellular level in this study.

### 2.3. Tissue Modeling

A two-dimensional (2D) cardiac tissue was represented by a rectangular grid of 225 × 225 points. Each point was regarded as a myocyte/Fbs complex. Fbs were coupled with each myocyte in two ways (longitudinal connection and lateral connection). Fbs distributed along the long axis of myocytes were referred as longitudinal connection, and along the short axis of myocytes was lateral connection. The electrical component was governed by the parabolic partial differential monodomain equation. The governing equations for the mechanical component were the stress equilibrium equations based on the large deformation theory [6]. The mechanical model proposed by Nash and Panfilov [34] was used, which included the stress equilibrium, the finite element approximations, the constitutive law, and the uniform isotonic boundary loads. 

 As the Fb-myocyte (F-M) ratio increases, the cardiac fibrotic remodeling leads to a progressive increase in cardiac passive stiffness [38, 39]. The common approach to modeling ischemic myocardium is to change material descriptions of passive myocardium. In this study, the material constants in passive material properties were reevaluated to show the higher elastic stiffness of fibrotic tissue. Compared with the normal tissue (the material constants with units of stress were 2 kPa and 6 kPa) [34], the material constants in fibrotic tissue were set to 4 kPa and 12 kPa, respectively. In this way, the modulus of fibrotic tissue was doubled, within the range of experiment results [40].

### 2.4. Numerical Methods

The system was solved numerically using custom software written in Fortran language. At the cellular level, all state variables were updated by the forward Eular method. The FDM was applied to solve the reaction-diffusion equation. Following each time integration step, all parameters of cells were updated. The active stress was then interpolated at the four-node rectangle isoparametric element Gaussian points. Stresses of these active Gaussian points were served as the inputs to the governing equations of the tissue mechanics model. The stress equilibrium equation was solved by a nonlinear least square iteration method with different material constants from different tissues. 

In the 2D coupling model, a tissue size of 225 × 225 grid points was used to study conduction velocity (CV) of plane wave propagation for longitudinal and transverse conduction. CV was calculated as the tissue length divided by the time interval between the time of stimulation and the time of the earliest excitation at the other end of the tissue. In this set of simulations, electromechanical activities and the change of strain due to a periodic point stimulation at the centre of the tissue were investigated. Central stimulus sites were chosen to be the smallest possible square regions (5 × 5 computational nodes), which made it possible to produce a propagating wave. Ten periodic central stimuli with a time interval of 500 ms were applied at the centre of the 2D tissue to ensure a stable excitation and contraction, and timing started when the 11th loop occurred. *I*_sac_ has been considered. For FDM, the spatial resolution in the longitudinal direction was 0.1 mm, while in the transverse direction was 0.04 mm. The temporal resolution was 0.005 ms. No-flux boundary conditions were used. For FEM, a mesh with 14 × 14 elements was used, which contained 16 × 16 grid points in plane wave and central point stimulus simulations. The temporal resolution for the mechanical model was 2 ms. Strain maps of central stimulus were also investigated.

## 3. Results

### 3.1. Effects of *I*_sac_ and Fbs on Atrial Myocyte AP

**Figure 1:**
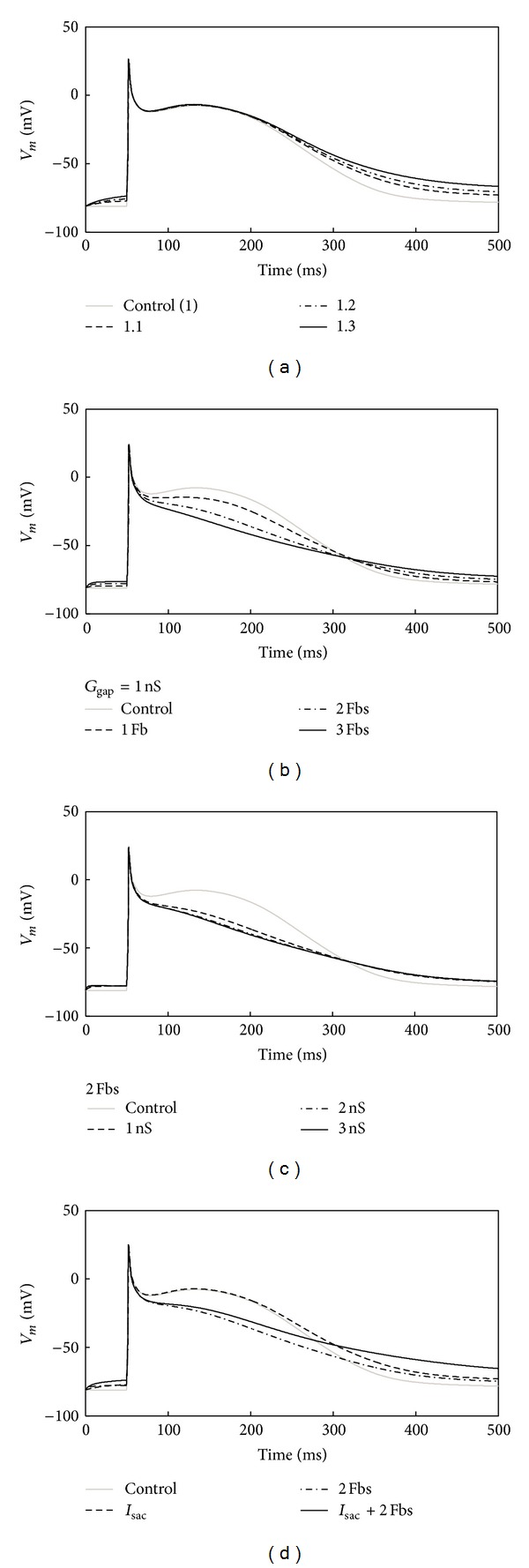
Illustration of changes in the waveform of the atrial myocyte AP. (a) 1 myocyte coupled to *I*_sac_ with a *λ* of 1.1 to 1.3. (b) 1 myocyte coupled to 1 to 3 Fbs with a *G*_gap⁡_ of 1 nS. (c) 1 myocyte coupled to 2 Fbs with a *G*_gap⁡_ of 1 to 3 nS. (d) 1 myocyte coupled to *I*_sac_ or 2 Fbs or *I*_sac_ and Fbs at the same time.

Figure 1(a) illustrates the AP of the human atrial myocyte coupled with *I*_sac_ for different stretch ratios *λ* = 1.1, 1.2, and 1.3 in comparison with the uncoupled control (*λ* = 1.0). With increasing *λ* of 1.0, 1.1, 1.2, and 1.3, prolonged repolarization was observed. Their corresponding AP durations at 50% repolarization (APD_50_) were 189 ms, 196 ms, 197 ms, and 202 ms. When compared with control (*λ* = 1.0), an increase of 3.7%, 4.2%, and 6.9% was obtained. At 90% repolarization, more obvious increase was found. AP durations (APD_90_) were 372 ms and 435 ms for *λ* = 1.1, 1.2, with the increase of 21% and 42% in comparison with 307 ms from *λ* = 1.0. With *λ* = 1.3, the membrane potential was −64.2 mV at 500 ms, and it did not return to −69.5 mV required for APD_90_. For the uncoupled control, the resting potential remained at −81 mV before the fast depolarization. For *λ* = 1.1, 1.2, and 1.3, it depolarized slowly within 50 ms and increased by 4.9%, 6.2%, and 8.6% at 50 ms when compared with the uncoupled control. Therefore, it can be concluded that the coupling *I*_sac_ prolonged repolarization and APD and depolarized the resting potential.

Figure 1(b) presents the repolarization for the human atrial myocyte when coupled with different numbers of Fbs (1 Fb, 2 Fbs, and 3 Fbs) in comparison with the control (no Fb). With more coupled Fbs, the membrane potential during the plateau was less depolarized, leading to a 13%, 41%, and 59% decrease (for 1, 2, and 3 Fbs) of APD_50_ when compared with control. However, for APD_90_, they were increased by 11%, 19%, and 45%. For the resting potential within 50 ms, it increased by 2.5–6.2% as the number of coupled Fbs increased. Therefore, coupling Fbs decreased APD_50_, prolonged APD_90_, and depolarized the resting potential. 

 Similar changes were observed in myocyte AP with changed *G*_gap⁡_ but with fixed number of coupled Fbs. As shown in Figure 1(c), increasing *G*_gap⁡_ from 1 ns to 3 ns resulted in a 43–55% reduction of APD_50_, an 18% increase of APD_90_, and a 5% increase in the resting potential at 50 ms.

Figure 1(d) shows the APs of four different coupling schemes. An uncoupled myocyte was referred to as control. The other three schemes were myocyte coupled with *I*_sac_ with *λ* = 1.1, myocyte coupled with two Fbs with *G*_gap⁡_ = 1 nS, and myocyte coupled with both *I*_sac_ and two Fbs with *λ* = 1.1 and *G*_gap⁡_ = 1 ns. It can be seen that *I*_sac_ made the membrane potential during the plateau slightly more depolarized, while Fbs made it less depolarized. For APD_50_, the first scheme resulted in a 5% increase and the second scheme resulted in a 41% decrease when compared with the uncoupled control. When the myocyte was coupled with *I*_sac_ and Fbs together, it was reduced by 38%. For APD_90_, coupling only with *I*_sac_ or Fbs produced a 21% and 19% increase. However, for the case of *I*_sac_ and Fbs together, the membrane potential was −62.5 mV at 500 ms, higher than the required threshold for APD_90_. In addition, the resting potentials within 50 ms were all depolarized in the four coupling schemes. The case of coupling with both *I*_sac_ and Fbs had the highest increase of 9.8% when compared with the control. The increases in other two couplings were ~5.5%.

### 3.2. Effects of *I*_sac_ and Fbs on Atrial Myocyte *T*_*a*_

**Figure 2:**
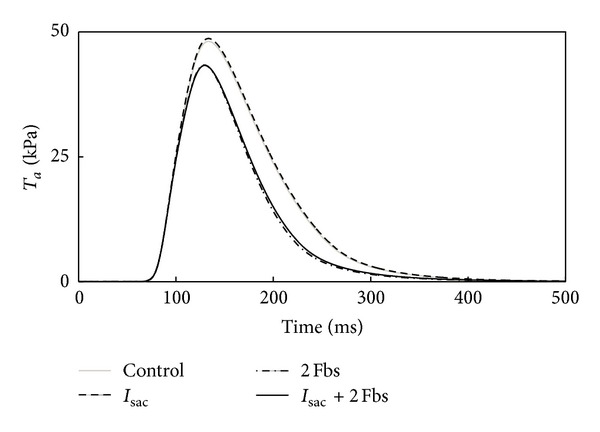
Illustration of changes in *T*_*a*_ of the atrial myocyte after coupling to *I*_sac_, 2 Fbs, or *I*_sac_ and Fbs at the same time.

As shown in Figure 2, with the same coupling schemes as described in Figure 1(d) and with the cells stimulated at 1 Hz, when compared to control, the peak of *T*_*a*_ was slightly decreased by the coupled *I*_sac_ and decreased by 11% with the coupled Fbs. Coupling *I*_sac_ and Fbs together did not result in a further decrease in the peak in comparison with that from coupling Fbs only. In addition, coupling Fbs (whether coupling *I*_sac_ or not) accelerated the reduction of *T*_*a*_. 

### 3.3. Coupling Fbs Modulate Conduction Velocity

**Figure 3:**
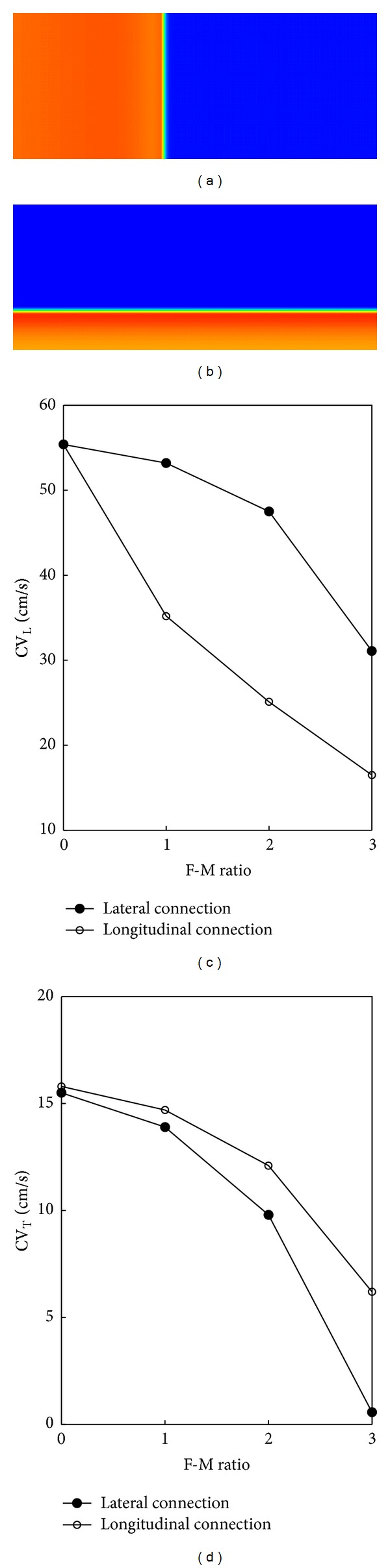
Effects of myocyte-Fbs coupling on CV: (a) longitudinal plane wave propagation, (b) transverse plane wave propagation. (c) CV_L_ versus F-M ratio for longitudinal and lateral connections, and (d) CV_T_ versus F-M ratio for longitudinal and lateral connections.

As shown in Figure 3, with the stimulation applied at the left end (for longitudinal conduction) or the bottom (for transverse conduction) of the tissue, it was observed that CV progressively decreased as the F-M ratio increased in either case of FMC. For longitudinal connection, Fbs were coupled at both ends of the myocyte, namely, Fbs distributed along the long axis. In this situation, with the F-M ratio from 1 to 3, longitudinal CV (CV_L_) decreased by 37% to 70%, and transverse CV (CV_T_) decreased by 7% to 61%. For lateral connection, Fbs were coupled along the lateral sides of a myocyte, with corresponding CV_L_ decreased by 4% to 44%, and CV_T_ decreased by 10% to 96%. From Figures 3(c) and 3(d), it can be seen that longitudinal connection resulted in a larger reduction in CV_L_ than in CV_T_, while lateral connection had a greater impact on CV_T_.

### 3.4. 2D Simulation with Central Stimulus

**Figure 4:**
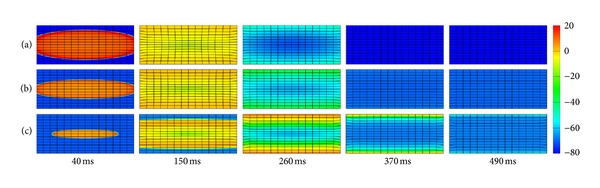
Central stimulus simulation of electromechanical coupling: (a) myocyte cells only, (b) 1 myocyte coupled to 2 Fbs with lateral connection in each grid point, and (c) 1 myocyte coupled to 3 Fbs with lateral connection in each grid point.

 Each grid in Figure 4(a) represents one atrial myocyte. Figure 4(b) gives one myocyte coupled with 2 Fbs (with an F-M ratio of 2). Figure 4(c) represents one myocyte coupled with 3 Fbs (with an F-M ratio of 3). *G*_gap⁡_ in Figures 4(b) and 4(c) was 1 nS. The type of FMC was lateral connection. 

In electrophysiology, it can be seen that the depolarization wave was generated in the center and spread out over time. The shape of the start of depolarization was ellipsoid. As the number of coupling Fbs increased, the conduction of excitation wave slowed down. At 40 ms, CV_L_ from 2 Fbs (Figure 4(b)) declined slightly when compared with the control (Figure 4(a)), but it declined significantly with 3 Fbs (Figure 4(c)). CV_T_ declined gradually as the F-M ratio increased, which was consistent with the results in Figure 3. Coupling with Fbs produced flat depolarization wave. As the deceleration of excitation wave, the myocytes coupled with Fbs required more time to return to the resting state. At 370 ms, tissues without Fbs and with an F-M ratio of 2 were at the resting state. However, the tissue with an F-M ratio of 3 did not reach the resting level even at 490 ms. In addition, with the contribution from *I*_sac_ and Fbs, the resting potential in tissues coupled with Fbs was less negative than that in the uncoupled tissue. 

In mechanics, with the *I*_sac_ and Fbs added in the tissue, the excitation in tissues was delayed, leading to the delay of *T*_*a*_ in the same grid point in comparison with the uncoupled control. As a result, the deformation in coupled tissues lagged behind. At 150 ms, the contraction was obvious in Figure 4(a), while imperceptible with 3 Fbs (Figure 4(c)). At 370 ms and 490 ms, meshes in Figures 4(a) and 4(b) gradually returned to relaxation along with the disappearance of propagating waves but kept contraction in Figure 4(c).

**Figure 5:**
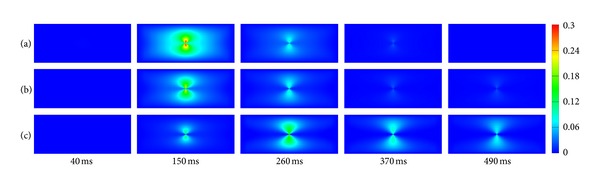
Strain maps of the central stimulus simulation: (a) myocyte cells only, (b) 1 myocyte coupled to 2 Fbs with lateral connection in each grid point, and (c) 1 myocyte coupled to 3 Fbs with lateral connection in each grid point.

Figure 5 shows the quantitative deformations corresponding to the electromechanical activities in Figure 4. It shows that higher strains appear in the center area. In the uncoupled tissue, the maximum strain was 0.28 at 150 ms. When the F-M ratio was 2 and 3, it declined to 0.22 and 0.17, decreased by 21% and 39%. As the F-M ratio increased, it took longer to reach the maximum. As shown in Figure 5(c), due to the delay of *T*_*a*_, the strain reached the peak value at 260 ms, 110 ms later than Figures 5(a) and 5(b). In addition, due to the slow conduction and less negative resting membrane in tissues with Fbs, there were still slight deformations presented in these tissues at 490 ms. Therefore, it can be concluded that coupling *I*_sac_ and Fbs delayed the deformation and decreased the maximum strain.

## 4. Discussion

The effects of coupling Fbs and *I*_sac_ on modulating human atrial myocyte excitability and AP morphology at the cellular level, and their effect on cardiac excitation wave conduction and contraction at the tissue level, have been investigated. The classical CRN model [30], the Rice model of the cardiac MF [32], the Maleckar model of atrial Fbs [33], and the Kuijpers model of *I*_sac_ [31] were employed in this study. All these models were integrated as a myocyte-*I*_sac_-Fb coupling electromechanical model to investigate AP waveform and *T*_*a*_ of human atrial myocyte as a function of *λ*, *G*_gap⁡_, and the number of Fbs. For tissue models, the influence of Fbs on plane wave propagation has been studied with the electromechanical activities and strain maps provided. 

### 4.1. Role of *I*_sac_ and Fbs Coupling on Human Atrial Myocyte Resting Membrane Potential, AP Waveform, and *T*_*a*_

For *I*_sac_, Zabel et al. explored the electrophysiological effect of sustained stretch and reported that a sustained, static load of the isolated rabbit ventricle influences repolarization and activation. The disparity of stretch effects between various locations in the ventricle resulted in an increased dispersion of repolarization [41]. Franz et al. confirmed that atrial SAC was involved in the pathophysiology of atrial fibrillation [42, 43]. In this study, membrane potential of the myocyte coupled to *I*_sac_ with different *λ* was investigated with a prolongation of repolarization and APD and a depolarization of the resting potential for *I*_sac_ coupling. Our results agreed well with the observation from Shaw and Rudy that increasing K^+^ concentration led to a depolarized membrane potential [44]. 

 For Fbs, experimental research showed depolarization of neonatal rat ventricular cardiomyocyte strands when myofibroblasts interacted with myocytes [25]. Previous studies of FMC using myocyte-Fb coculture models have shown that the Fbs depolarized electrotonically coupled myocytes [1, 25, 33, 45]. MacCannell et al. showed that the resting membrane potential of the coupled myocyte was depolarized slightly (~2.7 mV) for up to 10 Fbs per myocyte and was insensitive to changes in *G*_gap⁡_ [1]. In the study by Maleckar et al. a *G*_gap⁡_ of 8 nS and two active 1 Fbs resulted in a resting membrane potential elevation of 8.3 mV [33]. Our simulations also showed a depolarizing effect of coupled Fbs on resting membrane potential of the atrial myocytes. A maximum depolarization of 5.1 mV was obtained for an Fb density of 2 Fbs per myocyte with a *G*_gap⁡_ of 1 nS and a maximum depolarization of 3.9 mV for a high *G*_gap⁡_ (3 nS) with an F-M ratio of 2. 

 Coupling the human atrial myocyte with Fbs in this study also resulted in diverse effect on AP morphology during repolarization. Maleckar et al. used the FMC model with a high *G*_gap⁡_ and found that Fbs functioned as strong current sources at rest and as both sources and sinks during the AP [33]. In their study, the prolongation of repolarization was early in the AP, and plateau was prolonged or shortened depending on both the Fb resting membrane potential and number of coupled Fbs [33]. MacCannell et al. compared ventricular AP when Fbs of 6 pF or 60 pF were coupled to a myocyte. The data showed that APD was shortened much more when large Fbs were coupled [1]. In our simulations, a myocyte coupled with different number of Fbs or different *G*_gap⁡_ resulted in less depolarization in the membrane potential during the plateau when compared with control. Meanwhile, increasing Fbs and *G*_gap⁡_ decreased APD_50_ and prolonged APD_90_. 

 Myocyte-*I*_sac_-Fb coupling has been considered to investigate the resting membrane potential and AP waveform of human atrial myocyte as a function of *λ*, *G*_gap⁡_, and the number of Fbs. Compared with only Fbs coupling or *I*_sac_ coupling, the myocyte-*I*_sac_-Fb coupling had the highest depolarization at the resting membrane potential. However, the reduction of APD_50_ in myocyte-*I*_sac_-Fb coupling was between other two couplings. 

 In addition to the effect on the human atrial myocyte electrophysiology, coupled Fbs and *I*_sac_ had important implications for the changes in *T*_*a*_. In our study, *T*_*a*_ had the maximum value of 48.6 kPa, which was consistent with the normal range (10–75 kPa) [46]. Kerckhoffs et al. computed myofiber contraction in both nonfailing and failing hearts. Their results showed that in the failing heart model, inotropy was decreased by reducing peak fiber *T*_*a*_ by 27% [14]. In our study, the peak of *T*_*a*_ was decreased 11% with the coupled Fbs. The reason was that Fbs coupling reduced the Ca concentration and caused a reduction in *T*_*a*_. Due to the strong correlation between fibrosis and heart failure [47–49], it can be expected that once the number of Fbs increased, a significant reduction would be revealed in *T*_*a*_. In contrast, *I*_sac_ coupling did not bring much change in *T*_*a*_.

### 4.2. Role of Fbs Coupling on CV

In an experimental study, Miragoli et al. found a biphasic effect on CV when endogeneous Fbs proliferated with myocytes [25]. In a modeling study, Xie et al. showed that Fbs, whether coupled to myocytes or not, slowed conduction by creating zigzag conduction pathways [23]. In our simulation, CV_L_ and CV_T_ were investigated in both longitudinal and lateral connections. The results revealed an unidirectional decreasing in CV with Fbs coupling, which was consistent with Xie et al. [23]. However, different coupling types had different influence on CV_L_ and CV_T_. For longitudinal connection, the conductance along the longitudinal direction dropped down, resulting in a significant decrease in CV_L_. Similarly, lateral connection decreased the conductance along the transverse direction, which led to a higher reduction in CV_T_. 

### 4.3. Effects of *I*_sac_ and Fbs Coupling at the Tissue Level

For central stimulus simulations, the effect of Fbs on cardiac excitation conduction and contraction has been investigated in this study. Nash and Panfilov presented the same electromechanical activity due to a periodic point stimulation at the center of the excitable medium [34]. Their modeling did not consider the biophysics of specific ionic currents and SAC channels. Meanwhile, *T*_*a*_ has been directly modeled using a single ODE dependent on the transmembrane potential. In our study, specific cell electrophysiological and mechanical models of myocyte and Fb were included in the electromechanical modeling. Results in Figure 4 revealed that Fbs and *I*_sac_ coupling slowed down the conduction of excitation wave and depolarized the resting membrane potential, which confirmed results at the cellular level (Figure 1). Furthermore, due to the higher elastic stiffness of myocardial scar tissue [38–40], deformations in tissues coupled Fbs decreased by 21–39% when compared with uncoupled control (Figure 5). Our strain maps were similar to previous modelings and clinical data [10, 50]. In [50], strains in normal regions were −15.3 ± 4.5% and −10.6 ± 5.3% in the infarct zone. Our calculation of strain was also in this range. On the basis of these results, we expected that once severe fibrosis occurred, strains decreased apparently due to the large stiffness in these areas, or similar to the rigid motion.

### 4.4. Limitations

Three limitations of this study should be mentioned. Firstly, the mechanisms of Fb mechanics should be further studied. It can be expected that once the detailed mechanical model of Fb is developed, it can be coupled to the myocyte mechanics model to simulate more precise mechanisms of myocyte-*I*_sac_-Fb electromechanical coupling. Secondly, mechanosensitive currents have not been incorporated into the Fb model. However, previous studies implied that small electrical or mechanical perturbations in the cardiac Fb can alter the AP profile of the myocyte to which Fbs were coupled [51–53]. Thirdly, due to the lack of physiological data from the same species for the coupled model, only the existing experimental data relevant to each individual model was used to validate our simulation results. 

## 5. Conclusions

In conclusion, a coupled myocyte-*I*_sac_-Fb electromechanical model has been developed by integrating an active Fb model with a human atrial myocyte electrophysiological model (including *I*_sac_) and a mechanical model. The effects of Fbs and *I*_sac_ coupling on cardiac excitation conduction and contraction have also been investigated. The simulation results confirmed that Fbs and *I*_sac_ coupling resulted in diverse effects on AP morphology during repolarization, depolarized the resting membrane potential of human atrial myocyte, slowed down wave propagation and decreased strains in fibrotic tissue, and indicated that Fbs and SACs are important mechanisms in electromechanical coupling and should be considered in cardiac electromechanical modeling.
